# A Systematic Review of the Statistical Methods Adopted for Analyzing Follow-Up Data in Cohort Multiple Randomized Controlled Trial

**DOI:** 10.7759/cureus.51558

**Published:** 2024-01-03

**Authors:** Hina Narzari, Nilima Nilima, Venugopalan Y Vishnu, Maroof A Khan, Anu Gupta, Vasantha Padma Srivastava

**Affiliations:** 1 Biostatistics, All India Institute of Medical Sciences, New Delhi, New Delhi, IND; 2 Neurology, All India Institute of Medical Sciences, New Delhi, New Delhi, IND

**Keywords:** intention-to-treat, instrumental variable, cace, twics, cmrct

## Abstract

Background: The cohort multiple randomized controlled trial (cmRCT) can tackle some of the weaknesses of an RCT which has triggered the interest of researchers considerably over time. Several challenges persist regarding the methods of analyzing such valued data. The paucity of international recommendations concerning the statistical methods for analyzing trial data has led to a variety of strategies further complicating the result comparison. Our aim was to review the different cmRCT analysis methods since cmRCT was first proposed in 2010.

Methodology: A search for full-length studies presenting statistical analysis of the data collected adopting a cmRCT design was conducted on PubMed, Cochrane Library, EMBASE, JSTOR, Scopus, MEDLINE, and ClinicalTrials.gov.

Results: Out of 186 studies screened, we selected 22 for the full-text screening and 11 were found eligible for data extraction. All 11 studies were conducted in high-income countries, reflecting the design being underutilized in other settings. All of the studies were found to have used intention-to-treat (ITT) analysis with four of them utilizing instrumental variables (IV) analysis or a complier average causal effect (CACE). Randomization was noted often to be interchangeably used for random selection. Sample size calculation was not clearly specified in the majority of the studies.

Conclusion: Clarity regarding the distinction between an RCT and a cmRCT is warranted. The fundamental difference in design, which leads to certain biases that need to be taken care of by adopting IV or CACE analysis, has to be understood before taking up a cmRCT.

## Introduction and background

Among epidemiological study designs, randomized controlled trials (RCTs) lie at the top of the hierarchy in evaluating and efficiently translating research data into clinical practice. A study utilizing RCT design is known to be at the peak of evidence-based medicine due to its reliability [1]. However, missing the mark to meet recruitment targets, higher cost and attrition rate, and possibilities of response bias are some major drawbacks of an RCT [2]. To tackle some of these weaknesses of RCT, the Cohort Multiple RCT (cmRCT), also known as the Trials within Cohorts (TwiCs) design, was proposed by Clare Relton and colleagues in 2010 [3].

The cmRCT is a pragmatic trial design in which a large observational cohort is recruited comprising individuals fulfilling inclusion criteria. The number to be recruited is scientifically calculated. While enrolling into the cohort, consent from each participant is obtained. The established cohort subsequently facilitates conducting multiple (k) RCTs by randomly selecting nk participants out of n [3]. The effect remains the same as conventional design because the selection is purely based on chance. After selection, the participants are informed about the intervention, and second-stage consent is taken from them. The remaining eligible participants who were not randomly selected will form the treatment as usual/no treatment group. The group receiving usual treatment is not provided any information regarding the intervention. Similarly, for other interventions, the entire process can be concurrently imitated [3].

In a randomized study consisting of a treatment group and a control group, four different categories of participants exist according to compliance. Compliers are participants who receive the treatment they were assigned to. Always-takers are those who always take up the intervention, regardless of the group they were assigned to. Never-takers are those who never take the treatment irrespective of the group. Defiers are the participants who always take the intervention they were not assigned. Intention-to-treat (ITT) compares all the participants who were assigned to the treatment regardless of their receipt of the treatment [4]. ITT does not consider the lapses in compliance between groups [5]. Also, when non-compliance increases, the intervention effect may be increasingly diluted when using ITT. Along with random assignment, the treatment received is something that should not be ignored. By design, in cmRCT, consent is taken after randomly selecting the participants, and those refusing the intervention remain in the intervention group. Therefore, non-compliance in the intervention group is higher in trials using the cmRCT design. Hence complier average causal effect (CACE) estimation is recommended for analysis as it considers only those compliers who were offered the treatment. It also provides a fair statistical comparison between the treatment group compliers and the control arm's potential compliers [6]. A CACE analysis approach is considered a robust statistical analysis plan as it adjusts for the possibility that considerable numbers of participants may decline the treatment post-randomization [1]. However, a major issue noted in CACE is that it is not possible to know the compliance status of participants in the control arm [5,7,8]. One possible way to solve this issue is by using the Instrumental variable (IV) approach, where treatment effect estimates are adjusted by considering the proportion of non-compliers [6]. IV analysis estimates the effect of receipt of treatment instead of the effect of an offer of treatment [9]. IV analyses could be applied to account for noncompliance and may provide a better estimate of the intervention effect than the ITT analyses in case of high non-compliance [10].

A study utilizing cmRCT is intricately crafted to overcome the drawbacks of an RCT, generating highly valued data capable of yielding meaningful insights in changing the current line of treatment or denying a non-beneficial intervention. It overcomes the challenges of a pragmatic RCT by making the recruitment process faster and more efficient [11]. Again, keeping the “treatment as usual” group unaware of the intervention does not disappoint the participants (for reasons of not receiving the intervention), which results in lower attrition rates, very less possibilities of crossover, and leads to almost no chance of response bias [11,12]. The trial population in a cmRCT is broadly similar to the general population of patients [12]. The capability of a cmRCT has triggered the interest of researchers considerably over time with around 22 studies before and 291 after 2015. In conjunction with the increase, methodological advances were also observed, such as standardization of the design and adoption of standard statistical analysis. The progress permitted a not-so-distinct way of reducing the missing data and increasing compliance. However, several challenges still persist regarding the methods of analyzing such valued data. A weaker strategy for analyzing the data obtained will not only affect the findings of the well-designed study but will also under-utilize the available resources. Despite a few international recommendations [3,11,12] concerning the statistical methods for analyzing cmRCT data, most studies are noted to follow the strategy of an RCT. A high variability of analysis strategy was noted in similar trials, making comparison of results difficult. The present review aims to study the different cmRCT analysis methods used in trials since 2010 to date with no restriction on the type of intervention and participants.

## Review

Methods

The choice of studies is guided by the methodology utilized in this review. We reported this systematic review according to PRISMA 2020 guidelines [13]. Two reviewers (HN and NN) assessed all studies identified with a pre-defined checklist of criteria, including design (randomization, consent, number of intervention tested), sample size (cohort, RCT), data (rejection rate, outcome, follow-up duration, time points, attrition, missing data handling), and statistical techniques. In case of disagreement, the reviewers HN and NN resolved the conflicts via discussion.

PubMed, Cochrane Library, EMBASE, JSTOR, Scopus, MEDLINE, and ClinicalTrials.gov were searched comprehensively to identify articles appropriate to our study. A search strategy was developed by going through the search guidelines of all the databases thoroughly. A combination of words such as “cmRCT,” “Cohort multiple randomized controlled trial,” “TwiCs,” and “Trials Within Cohorts” were used to capture studies that have used cmRCT as their study design. Appendix contains the complete search strategy used.

All the citations and abstracts retrieved were then imported. Duplicate articles were removed, and the remaining articles were assessed for inclusion by two researchers (HN and NN). Initially, our search was limited to studies published in the English language from January 1, 1990 to August 22, 2023, but since cmRCT was proposed in the year 2010, we checked the articles separately from 1990 to 2009 and found them irrelevant to our study hence changed our search date to May 1, 2010, to August 22, 2023. Articles were excluded if they were registered in clinicaltrials.gov but had no publications, study design followed was RCT and not cmRCT or, were review articles concerning cmRCT. Articles that were erratum or reply to other articles and studies that published only the protocol and not the results were also excluded from this systematic review. Feasibility studies that were done to understand the barriers to conduct were also excluded. Pilot studies are often conducted with a small sample size to assess the feasibility and difficulties faced. Hence, judging a pilot study analysis for its appropriateness may be superfluous. Thus, pilot studies were not considered for full-text screening. In the abstract screening, conference proceedings were also excluded where abstracts were available, but no full-text article could be found. Along similar lines, articles included in full-text screening were assessed for final inclusion. Studies that fulfilled all of the inclusion criteria were included in this review.

Results

**Figure 1:**
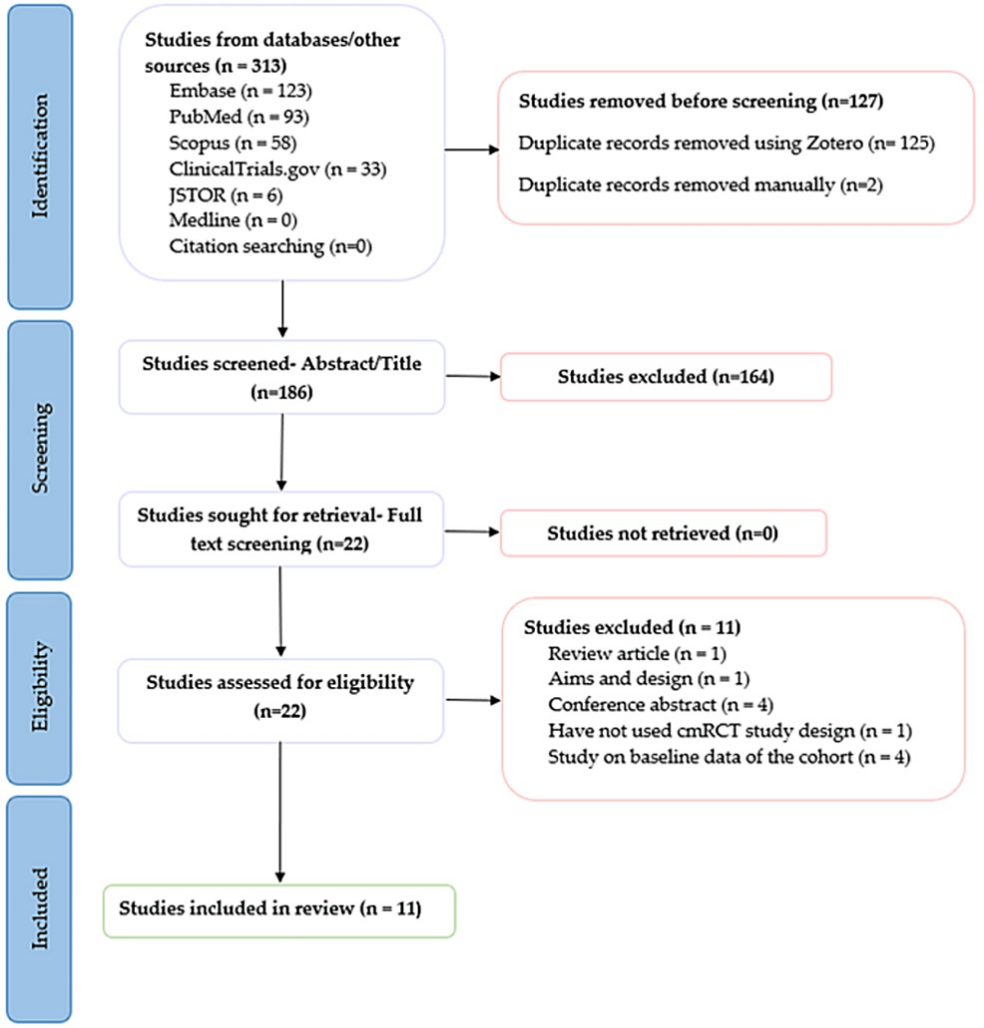
PRISMA diagram of the flow of articles through various stages of data extraction based on the pre-defined criteria.

**Figure 2:**
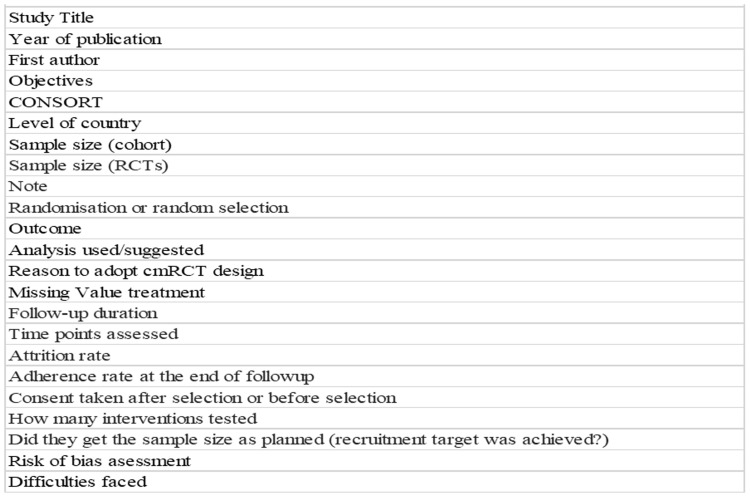
Checklist used for the extraction of data from articles included in our study.

**Figure 3:**
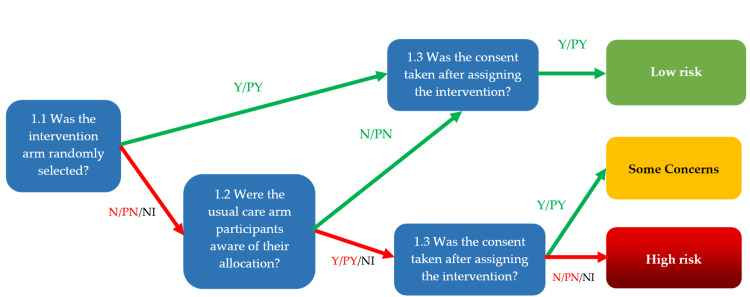
Algorithm for suggested judgement of risk of bias arising from the random selection process

**Figure 4:**
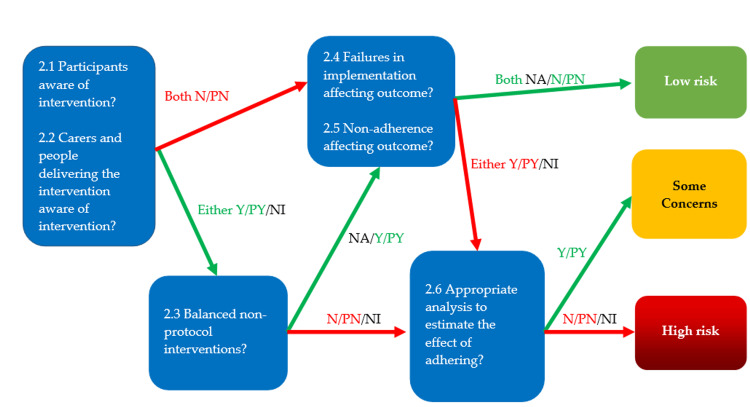
Algorithm for suggested judgement of risk of bias due to deviations from the intended interventions (effect adhering to the intervention)

The database search, it yielded 313 records. After the removal of 127 duplicates, 186 articles remained for abstract screening. Upon exclusion of 164 studies that did not fulfil the inclusion criteria, 22 publications were identified as eligible for full-text screening based on their presentation in the abstract and the title. Full-text screening of 22 articles led to the exclusion of 11 articles [10,12,14-21]. Figure 1 presents the PRISMA diagram of the flow of articles. After a full-text review, the relevant articles were taken to the data extraction stage, where the tables were populated based on the pre-defined checklist of criteria. Articles were then assessed for the risk of bias and seven articles had some concern and four articles had high risk. The checklist is presented in Figure 2 in Appendix and modified RoB2 is presented in Table 1 and described in Appendix, Figures 3, 4.

**Table 1:** Modified Risk of Biased Assessment Tool for included full text studies in the presented review Y - Yes; PY - Probably Yes; N - No; PN - Probably No; NI - No information

Articles	Petit et al., 2023	Jolliffe et al., 2022	Panagioti et al., 2018	Couwenberg et al., 2020	Viksveen et al., 2017	Gal et al., 2021	Williams et al., 2018	Dupuis et al., 2023	Kwakkenbos et al., 2022	Pielkenrood et al., 2022	Fahim et al., 2022
Risk of bias arising from the randomization process											
1.1 Was the intervention arm randomly selected?	PY	N	Y	N	Y	N	N	N	N	N	N
1.2 If N/PN/NI in 1.1: Were participants in the usual care arm aware of their assigned group?	-	Y	N	Y	N	N	Y	Y	N	N	Y
1.3 Was the consent taken after assigning the intervention	PY	Y	Y	Y	Y	Y	N	PN	Y	Y	Y
Risk-of-bias judgement	Low	Some Concerns	Low	Some Concerns	Low	Low	High	High	Low	Low	Some Concerns
Risk of bias due to deviations from the intended interventions (effect of adhering to intervention)											
2.1. Were participants aware of their assigned intervention during the trial?	Y	Y	Y	Y	Y	Y	N	PY	Y	Y	Y
2.2. Were carers and people delivering the interventions aware of participants' assigned intervention during the trial?	Y	Y	Y	Y	Y	Y	Y	Y	Y	Y	Y
2.3. [If applicable:] If Y/PY/NI to 2.1 or 2.2: Were important non-protocol interventions balanced across intervention groups?	Y	Y	Y	Y	Y	Y	Y	Y	Y	Y	Y
2.4. [If applicable:] Were there failures in implementing the intervention that could have affected the outcome?	N	N	N	N	N	N	N	N	N	N	Y
2.5. [If applicable:] Was there non-adherence to the assigned intervention regimen that could have affected participants’ outcomes?	N	N	Y	N	Y	Y	N	Y	Y	Y	N
2.6. If N/PN/NI to 2.3, or Y/PY/NI to 2.4 or 2.5: Was an appropriate analysis used to estimate the effect of adhering to intervention?	-	-	Y	-	Y	Y	-	N	Y	N	-
Risk-of-bias judgement	Low	Low	Some Concerns	Low	Some Concerns	Some Concerns	Low	High	Some Concerns	High	Low
Risk of bias due to missing outcome data											
3.1 Were data for this outcome available for all, or nearly all, participants randomized?	NI	N	Y	NI	N	N	N	Y	N	N	NI
3.2 If N/PN/NI to 3.1: Is there evidence that the result was not biased by missing outcome data?	N	Y	-	N	N	N	Y	-	N	Y	N
3.3 If N/PN to 3.2: Could missingness in the outcome depend on its true value?	Y	-	-	N	N	N	-	-	N	-	N
3.4 If Y/PY/NI to 3.3: Is it likely that missingness in the outcome depended on its true value?	N	-	-	-	-	-	-	-	-	-	-
Risk-of-bias judgement	Some Concerns	Low	Low	Low	Low	Low	Low	Low	Low	Low	Low
Risk of bias in measurement of the outcome											
4.1 Was the method of measuring the outcome inappropriate?	N	N	N	N	N	N	N	N	N	N	N
4.2 Could measurement or ascertainment of the outcome have differed between intervention groups?	N	N	N	N	N	N	N	N	N	N	N
4.3 If N/PN/NI to 4.1 and 4.2: Were outcome assessors aware of the intervention received by study participants?	Y	Y	Y	Y	PN	Y	N	NI	Y	NI	N
4.4 If Y/PY/NI to 4.3: Could assessment of the outcome have been influenced by knowledge of intervention received?	N	N	N	PY	-	N	-	N	N	N	-
4.5 If Y/PY/NI to 4.4: Is it likely that assessment of the outcome was influenced by knowledge of intervention received?	-	-	-	Y	-	-	-	-	-	-	-
Risk-of-bias judgement	Low	Low	Low	High	Low	Low	Low	Low	Low	Low	Low
Risk of bias in selection of the reported result											
5.1 Were the data that produced this result analysed in accordance with a pre-specified analysis plan that was finalized before unblinded outcome data were available for analysis?	Y	PY	Y	PY	Y	Y	N	Y	Y	Y	Y
Is the numerical result being assessed likely to have been selected, on the basis of the results, from...											
5.2. ... multiple eligible outcome measurements (e.g. scales, definitions, time points) within the outcome domain?	N	N	N	N	N	N	N	N	N	N	N
5.3 ... multiple eligible analyses of the data?	N	N	N	N	N	N	N	N	N	N	N
Risk-of-bias judgement	Low	Low	Low	Low	Low	Low	Some Concerns	Low	Low	Low	Low
Overall Risk-of-bias judgement	Some Concerns	Some Concerns	Some Concerns	High	Some Concerns	Some Concerns	High	High	Some Concerns	High	Some Concerns

Petit [22] conducted a recent phase II RCT with a cohort of size 262, which is not specified to be scientifically calculated. A total of 253 patients were divided into four strata, and within each stratum, random selection was done to equally allocate among the intervention (n=125) and the control group (n=128). This study frequently used the term randomization and allocation instead of random selection against the recommended “random selection” in a cmRCT [3]. All randomized patients’ data were analyzed using the ITT. CACE or IV analysis was not used in this study.

Jolliffe [23] collected monthly data of 6,200 adults in the United Kingdom adults. A scientific calculation of sample size resulted in a total of 6,200 to be randomly selected to detect a 20% reduction in the proportion of participants meeting the primary outcome with 84% power and a 5% level of significance and accounting for the 25% loss to follow-up. The 6,200 were later randomized; each intervention arm comprised 1,550, and the control arm comprised 3,100, participants. Considering an 84% power and a consolidated 6,200 sample size raises a few concerns regarding the sample size calculation. Consent to be a part of the intervention was taken post-randomization. The median or mode value of covariate among participants with non-missing data was imputed in place of missing data of participants. Primary analyses were performed using ITT, with the final sample size being less than requisite. CACE or IV analysis was not used in this study. However, the sample size of 6,200 was obtained keeping in mind a lost to follow-up of 25%, making an effective sample size 4,650, which has been achieved with 5,636 out of 6,200 (90.9%) adhered to the intervention and 5,979 individual data utilized in ITT.

In 2018, Panagioti [24] conducted a study in United Kingdom on 1306 eligible participants from the PROTECTS cohort. The study was powered to 80%, 5% level of significance to detect a standardized effect size of 0.25, allowing for 25% attrition among participants, which indicated a sample size of 504 participants or 252 per arm. The 252 were randomly selected to be provided with the intervention but due to a lower uptake rate than anticipated, another 252 participants were included in the intervention group. The rest, 802 out of 1,306 eligible, were given usual care. Consent regarding the intervention was taken after selecting participants for the intervention arm. ITT principle was followed for analysis. Sensitivity analyses were run, and an estimate of the effect of treatment on patients consenting to treatment was derived using a CACE analysis. However, the authors have claimed a final sample size of 504 was obtained accounting for the 25% attrition and making the effective sample size 378 or 189 in each arm. It is noted that the number who consented to the intervention was 207, with 189 adhering to the intervention. Missing data values were imputed using multiple imputation chained equation.

Couwenberg [25] conducted a study on 60 patients per arm for a one-sided test α=0.15, and 80% power with an estimated proportion of participants of 30% in the intervention and 13% in the control arm achieving the primary outcome, an estimated 20% proportion of patients refusing the intervention. A one-sided test and higher α were used as is usually recommended for phase 2 screening trials. Noting a higher-than-anticipated refusal rate, the sample size was increased to 128. Patients were randomized, after which written informed consent was taken against the recommended “random selection” in a cmRCT. ITT analysis was performed on 64 individuals in each arm as calculated, including the 12 participants who did not consent and one who did not receive the intervention. Because they had adjusted for the 20% refusal rate while calculating the sample size, their effective sample size being 60, the minimum number required initially would have been 50, which is achieved. CACE or IV analysis was not adopted in this study.

Another study by Viksveen [26] anticipated an effect size of 0.35, alpha 0.05, power of 80%, and an anticipated non-response rate of 40%, resulting in a sample size of 162 and 323 in treatment and standard of care groups respectively. All 566 eligible participants were recruited in the cohort, and 185 were randomly selected for the intervention arm. However, the CONSORT flow diagram presents a disparity with the writeup. A clear CONSORT flow diagram is warranted. This was the first full trial of any intervention utilizing cmRCT. Hence, they have presented the findings using ITT as well as IV analysis with 74 participants consenting for the intervention and 103 responses analyzed through ITT, where an effective sample size of 98 in the intervention arm was necessary. The study had a very low response rate. Four approaches for dealing with missing data were used including no imputation for missing data, multiple imputation, regression imputation and last observation carried forward. MCAR test was used to check missing patterns.

Gal [27] noted that a sample of 260 patients would be necessary for an acceptance rate of 55% in the intervention group, and a clinically relevant 10-point difference in outcome 80% power and a 5% two-sided level of significance. Randomization was performed using a computer-generated randomization list with an allocation ratio of 1:1. The use of randomization in place of random selection is to be noted. However, consent for the intervention was taken after allocation into the intervention arm. ITT linear regression analysis was used to assess between-group differences; IV analysis was used to find the effect of intervention, and propensity score (PS) analysis was used to check the robustness of IV analysis. The effective sample size in the intervention arm was 91 (i.e., 70% of 130); 68 (52.3%) had accepted the offer, and ITT was performed on the data obtained from 110 participants. Missing values on covariates and baseline measures of the outcome were imputed through the multiple imputation method. The attrition rate was 13%, and 44.1% adhered to the intervention till completion.

Williams [28] conducted a study on 80 participants in each group, which was scientifically calculated, allowing for a 15% loss to follow-up, 90% power, and a two-sided alpha of 0.025 to detect a clinically meaningful difference of 1.5 units in pain intensity and a standard deviation of 2.3 units. The cohort size was 160 participants, who were then randomized using a central concealed random allocation process into intervention and control groups in a 1:1 ratio. Consent was taken before randomization. Outcomes were analyzed under the ITT principle. CACE and IV analysis were not used. Accounting for 15% lost to follow-up and a final sample size of 80 in each group, the effective sample size was 68 in each group, resulting in a total of 45 patients receiving the intervention. However, ITT was performed on 79 participants’ data. AUC was computed by interpolating the data on participants with <10% missing and using multiple imputation chained equation for those with >10% missing.

Dupuis [29] considered 131 adolescents with no specification if the sample size was calculated scientifically. However, they performed a post-hoc power calculation and reported a power of 80% to detect a difference in the primary outcome. The authors have adopted the block randomization technique instead of random selection. Two interventions were assigned to the eligible 26 and 51 participants, respectively, and another 51 participants received usual care. Consent for recruitment was obtained; however, they did not state clearly whether consent was obtained before or after randomization. An ITT approach was adopted to perform a complete case analysis followed by a sensitivity analysis. An ITT analysis was used. IV approach was not adopted for the data analysis. The authors were aware of the low and further decreasing sample size being a limitation of this study.

Kwakkenbos [30] conducted a study where for a standardized mean difference of 0.25, 5% level of significance, 80% power, and 10% loss to follow-up, a minimum of 586 participants were required to conduct the study. There was no clarity on whether 586 in each group is required, as reproducing the said sample size resulted in 279 in each group. However, 466 participants were randomized, of which 280 were allocated to the intervention and 186 to the usual arm, with 170 consenting to the intervention. Consent was taken post-randomization. ITT analysis was used to estimate differences in scores between 212 intervention and 151 control participants which is less than the minimum number required. To estimate average intervention effects among compliers, they used an IV approach. However, for future trials they plan to adopt an RCT and obtain consent before randomization in order to control for the low consent rate. A bootstrap resampling was used to obtain the 95% CI. Multiple imputation by chained equations was used to treat the missing data. The size of the sample of participants recruited was lower than calculated. The consent rate was low (60%).

Another study from the Netherlands, by Pielkenrood [31], did not perform a scientific sample size calculation. Eligible participants were equally randomized using the block randomization technique. The authors have failed to perform a random selection of eligible participants into the intervention arm; however, they claimed to have obtained consent post-random selection. A total of 55 participants were allocated to each arm. ITT and PP analysis was performed. Missing outcome data were assumed to be missing at random and analyzed accordingly. The authors believed their study to be negatively influenced by the drop-out after randomization in this trial.

A study by Fahim [32] was conducted among 163 participants who were then randomized to the intervention arm, followed by informed consent. For a one-day difference (five versus six days, intervention versus control) in the duration of hospital stay, a 10% refusal rate in the intervention arm, a two-sided type one error of 5%, power 80%, and a minimum of 82 eligible participants per arm were required. However, anticipating a loss in power due to utilizing non-parametric tests, they increased the sample size by 15% to 98. Subsequently, they decreased this to 75, removing the refusal rate of 10% due to almost no refusal in the intervention arm. Thus, 75 patients in each arm were found to be sufficient. The clarification on the standard deviation equivalent was missing from the writeup to reproduce the sample size calculation. ITT and PP analyses were used.

According to Relton, cmRCT is best suited to situations that do not require participants being blinded [3]. However, blinding of outcome assessor and statistical analyst is reasonable. Among the articles included in this study, Petit [22], Jolliffe, Panagioti [24], Couwenberg [25], and Gal [27] blinded no investigators, clinicians or patients during the trial. Viksveen [26] used blinding during the random selection process, but later, no blinding was used even for statistician doing the analysis because group allocation would become obvious from the randomization ratio provided. Williams [28] blinded the outcome assessors and statisticians to the treatment allocation. Dupuis [29] and Pielkenrood [31] provided no information regarding blinding. Kwakkenbos [30] blinded the statistical analysts to trial arm allocation, but were again unblinded for the complier effect analyses, which required the knowledge of intervention arm consent. Fahim [32] blinded the investigators who performed the analyses.

The articles included in our study were also carefully evaluated for their adherence to the CONSORT guidelines. It was seen that five out of 11 articles failed to adhere to a number of points in the guidelines. Petit [22] missed to mention the settings and locations from where the data was collected. Couwenberg [25] and Gal [27] did not mention whether the trial was generalizable or not. Further, we noticed Gal [27] did not completely define prespecified primary and secondary outcome measures; the article also did not mention how and when the outcomes were assessed. In another article, Dupuis [29] did not state how the sample size was determined, and no dates defining the periods of recruitment and follow-up were given. Pielkernrood [31] did not provide sufficient details of their experimental and control interventions to allow replication and how and when the intervention was administered. The sample size determination method was also found missing from this article. The other six articles [23,24,26,28,30,32] used the CONSORT reporting guidelines thoroughly.

Discussion

The present systematic review was conducted with the objective of summarizing and evaluating the current knowledge on the statistical analysis methods that should be adopted when utilizing a pragmatic design, namely a cmRCT. Out of 186 studies screened, 22 were selected for full-text screening, and 11 were found to be eligible for data extraction. The capability of a cmRCT has triggered the interest of researchers considerably in recent times with close to 22 studies before and 291 after 2015. Of the 11 full-length studies published with results showing an intervention effect, three were conducted before 2020 and eight in and after 2020, reflecting the considerable increase in interest of researchers over time. However, all 11 studies were conducted in high-income countries, reflecting the design being underutilized in other settings. Majority of the studies adopted the cmRCT design due to its ability to mimic the process of treatment decision-making in routine care, low attrition, efficient patient recruitment, generalizability of results, and decreased disappointment bias. Only one of the studies adopted cmRCT for its ability to partake in multiple interventions in the same cohort, apart from other advantages that a cmRCT offers. Sample size calculation was not clearly specified in the majority of the studies. The latter is an important aspect of data analysis and findings in any study. Randomization was noted to often be used interchangeably for random selection. Very few articles had thoroughly used random selection and taken consent post-selection. Though it is recommended that use random selection, most studies have failed to adhere to the guidelines and have used randomization instead. Studies must follow the blinding of the outcome assessor and statistician doing the analysis to rule out any form of bias in the data collection and interpretation. Few articles were found to have followed the blinding of the outcome assessor and the statistician doing the analysis.

The first publication presenting the statistical analysis of a cmRCT was in 2017 by a group of authors that included Clare Relton, who introduced the cmRCT design in 2010. All of the studies were found to have used ITT analysis, with very few utilizing IV or a CACE analysis. The attrition rate resulting in missing data often decreases the power of the statistical tests in making decisions. Assessing the missing pattern and utilizing the missing data treatment often helps retain the statistical tests’ power as anticipated during the sample size calculation. The majority of the studies were found to have used missing data treatment with detail in the data extraction sheet. In summary, this review identified and compared different methods for analyzing the data collected by adopting a cmRCT design. Different methods are appropriate depending on the refusal and non-compliance rate, which is highlighted to provide supervision regarding the application of these methods in practice. Further research on IV and CACE analysis in a cmRCT would be desirable to follow up on the results of this article. Despite several strengths, there is a limitation of this review, which includes the availability of limited full-length articles that have published analysis findings since the inception of the cmRCT design in 2010. Clarity on the distinction between an RCT and a cmRCT is warranted.

## Conclusions

The basic difference in design, which leads to certain biases that need to be taken care of by choosing to adopt IV or CACE analysis, has to be understood before taking up a cmRCT. The findings of our study should help researchers understand the choice of methods and identify the most appropriate analysis for cmRCT data. This is a prerequisite for a recommendation of an analysis plan in similar situations. Researchers with an ongoing or upcoming cmRCT will benefit from this review by adopting the appropriate analysis method, which in turn should improve decision-making.

## Appendices

Search Strategy

PubMed

"Cohort multiple randomized controlled trial"[All Fields] OR "trials within cohorts"[All Fields] OR "cmrct"[All Fields] OR "twics"[All Fields] OR “cohort multiple randomised controlled trial"[All Fields] OR "Trials within cohort"[All Fields] OR "cohort multiple randomised controlled trials"[All Fields] OR "cohort multiple randomized controlled trials"[All Fields] OR "trial within cohort"[All Fields]

Cochrane Library

“Cohort multiple randomized controlled trial*” OR “Cohort multiple randomised controlled trial*” OR “Trial* within cohorts” OR cmRCT OR TWiCs

Title Abstract Keyword label was selected to limit search to terms in the record title, abstract and keyword fields.

Embase

(“Cohort multiple randomi?ed controlled trial*”.ti,ab. or “Trial* within Cohorts”.ti,ab. or cmRCT.ti,ab. or TWiCs.ti,ab.)

JSTOR

(((("Cohort multiple randomized controlled trial*") OR ("Trial* within cohorts")) OR (cmrct)) OR (twics))

Scopus 

{Cohort multiple randomized controlled trial*} OR {Trial* within cohorts} OR cmrct OR twics

Medline

“Cohort multiple randomized controlled trial*”.ti,ab. or “trial* within cohorts”.ti,ab. or +cmRCT.ti,ab. or +TWiCs.ti,ab.

ClinicalTrials.gov.

cmrct OR twics OR "cohort multiple randomized controlled trial*" OR "trial* within cohorts"

Pre-defined checklist for data extraction

Risk of bias assessment

Risk of bias was assessed using the revised Cochrane risk of bias tool for randomized trial (RoB2) which has assessments concerning the randomization and allocation concealment. In the presented review, studies being assessed have followed a cmRCT design which is similar to an RCT with few modifications. To avoid disappointment bias among the control arm, a random selection is recommended in place of randomization, hence we have assessed the articles for random selection done or not (Section 1.1 of RoB2).  If random selection is not done, we will assess if the control/usual care arm were aware of their allocation. Hence, “1.2 If N/PN/NI in 1.1: Were participants in the usual care arm aware of their assigned group?" was added. If the participants in the control arm were aware of their allocation it would raise some concerns. Another signaling question “1.3 Was the consent taken after assigning the intervention” was added to the Risk of bias arising from the randomization process section. Consent taken after selection of participants into the intervention arm was considered to have low risk of bias while consent for intervention before assignment to the intervention arm was judged to have high risk of bias. Few sections (1.2, 1.3, effect of assignment of intervention) were removed as they were not applicable to the current context and design.
